# Respiratory Care for the Ventilated Neonate

**DOI:** 10.1155/2018/7472964

**Published:** 2018-08-13

**Authors:** Gustavo Rocha, Paulo Soares, Américo Gonçalves, Ana Isabel Silva, Diana Almeida, Sara Figueiredo, Susana Pissarra, Sandra Costa, Henrique Soares, Filipa Flôr-de-Lima, Hercília Guimarães

**Affiliations:** ^1^Department of Neonatology, Centro Hospitalar São João, Porto, Portugal; ^2^Faculty of Medicine, University of Porto, Porto, Portugal; ^3^Department of Physical and Rehabilitation Medicine, Centro Hospitalar São João, Porto, Portugal

## Abstract

Invasive ventilation is often necessary for the treatment of newborn infants with respiratory insufficiency. The neonatal patient has unique physiological characteristics such as small airway caliber, few collateral airways, compliant chest wall, poor airway stability, and low functional residual capacity. Pathologies affecting the newborn's lung are also different from many others observed later in life. Several different ventilation modes and strategies are available to optimize mechanical ventilation and to prevent ventilator-induced lung injury. Important aspects to be considered in ventilating neonates include the use of correct sized endotracheal tube to minimize airway resistance and work of breathing, positioning of the patient, the nursing care, respiratory kinesiotherapy, sedation and analgesia, and infection prevention, namely, the ventilator-associated pneumonia and nosocomial infection, as well as prevention and treatment of complications such as air leaks and pulmonary hemorrhage. Aspects of ventilation in patients under ECMO (extracorporeal membrane oxygenation) and in palliative care are of increasing interest nowadays. Online pulmonary mechanics and function testing as well as capnography are becoming more commonly used. Echocardiography is now a routine in most neonatal units. Near infrared spectroscopy (NIRS) is an attractive tool potentially helping in preventing intraventricular hemorrhage and periventricular leukomalacia. Lung ultrasound is an emerging tool of diagnosis and can be of added value in helping monitoring the ventilated neonate. The aim of this scientific literature review is to address relevant aspects concerning the respiratory care and monitoring of the invasively ventilated newborn in order to help physicians to optimize the efficacy of care.

## 1. Introduction

There is an increasing trend in the neonatal intensive care units (NICUs) to use noninvasive ventilation modes; however, invasive ventilation is still often necessary for treating preterm and term infants with respiratory insufficiency [1, 2].

Nowadays, very preterm infants are extubated relatively quickly, and prolonged invasive ventilation is considered an important risk factor for bronchopulmonary dysplasia (BPD) development [3].

Many different ventilation modes and strategies are available to optimize mechanical ventilation and to prevent ventilator-induced lung injury, and volume-targeted ventilation modes had reported better outcomes when compared to pressure-limited ventilation modes [4].

Important aspects to be considered in ventilating neonates include the use of correct sized endotracheal tube, positioning of the patient, the nursing care, respiratory kinesiotherapy, sedation and analgesia, and infection prevention, namely, the ventilator-associated pneumonia and nosocomial infection, as well as the treatment of complications such as air leaks and pulmonary hemorrhage [3, 5].

Aspects of ventilation in patients under ECMO (extracorporeal membrane oxygenation) and in palliative care are of increasing interest nowadays. Monitoring of the ventilated neonate using online pulmonary mechanics and function testing as well as capnography are becoming more commonly used, and echocardiography is now a routine in most neonatal units. Near infrared spectroscopy (NIRS) is becoming an attractive tool in helping to prevent neurological damage and lung ultrasound is an emerging tool of diagnosis and can be of added value in helping monitoring the ventilated neonate.

Individualized nursing care and respiratory therapy are crucial and have become increasingly widespread in NICUs. It has been demonstrated that each mechanically ventilated infant requires, on average, the care of one nurse during 60% of the time [6].

Our aim is to review the scientific literature concerning the respiratory care and monitoring for the invasively ventilated newborns in order to help physicians to optimize the efficacy of care while preventing deleterious iatrogenic effects.

## 2. The Endotracheal Tube

The endotracheal tube (ETT), the fundamental interface between patient and ventilator, is a cornerstone for successful ventilation. The appropriate sized tube could be defined as the one with the largest possible inner diameter (ID) that will comfortably pass through the vocal cords providing a sufficient seal for effective ventilation. Confirming the existence of a small leak at peak inspiratory pressures of ≥20 cm H_2_O could help prevent the use of an excessively large tube [7]. According to Poseuille's law, the resistance is inversely proportional to the fourth power of the tube radius (the tube ID), increasing as the tube ID decreases [8, 9]. Resistance is particularly marked for tubes with ≤2.5 mm ID, with smaller than expectable differences between the 3.0, 3.5, and 4 mm tubes [8]. Tube length also increases resistance, and unnecessarily long tubes should be cut [9]. Shouldered tubes have also been proved to offer less resistance (up to 50% less) and should be considered, particularly for the smaller ID tubes [9, 10]. Existing tables, derived from clinical and experimental studies, should guide initial tube size selection [11].

The neonatal airway has classically been described as differing from that of older children (>8 years) and adults with the narrowest part located at the cricoid cartilage [12]. Although recent findings have put it in question, this anatomical feature has guided years of practice with the use of uncuffed endotracheal tubes (UTT) for small children, infants, and neonates [13]. Since the development of high-volume low-pressure (HVLP) cuffs, cuffed endotracheal tubes (CTT) have been growingly used in smaller children, infants, and even neonates [14]. A 2016 report on current practice patterns of the American Society of Pediatric Anesthesia found that 60% of responders used CTT on full-term neonates >50% of the time [7]. Although several short-term reports have deemed CTT safe to use in neonates with no greater rates of complications than UTT (provided that cuff pressure is monitored) [15, 16], there have also been reports contradicting those findings [17, 18]. Data on the safety and efficacy of the use of CTT for prolonged ventilation and impact on longer-term outcomes, in the NICU setting, are lacking [14].

Few studies compared nasotracheal versus orotracheal intubation. Postextubation atelectasis may be more frequent after nasal intubation, particularly in extremely low birth weight (ELBW) neonates. No route has been proven preferable to the other, with similar rates of complications between them [19].

To clinically confirm endotracheal intubation, current resuscitation guidelines recommend a combination of clinical signs (breath sounds under the axillae, thoracic expansion, increased heart rate, and condensation visible on the tube) and exhaled CO_2_ monitoring. Although effective in most situations, they must be interpreted with care in the context of cardiac arrest [20].

After successful intubation, placing the tube at a correct depth is vital. Excessively deep insertion could lead to selective right main bronchus intubation, pneumothorax, and/or atelectasis. A shallow insertion puts the patient at greater risk for accidental extubation.

The ideal safe position for the ETT tip is the midtracheal position, usually at T1-T2 level [21–23]. In ELBW neonates, however, the level of the carina can be as high as T3, instead of the usual T4, which would place the midtracheal position slightly higher [23].

Several rules and formulas have been developed to help predict ideal tube length [24]. The commonly “7-8-9” rule, developed by Tochen [25], that in its simplified form could be translated as 6+ weight (for orotracheal intubation), assumes a linear relation between body weight and proper tube length. Peterson et al. [26] and Amarilyo et al. [27] questioned these findings for ELBW neonates, as the use of Tochen's formula would result in excessively deep insertions of ETT, in that subgroup. Kempley et al. [28] demonstrated a linear relation between ETT tube length with gestational age but not with body weight and proposed a model that used gestational age (presented in the form of table) for better ETT length prediction (Table 1). Flinn et al. [29] were however unable to find a significant difference between Kempley's gestational age table and Tochen's body weight formula for ETT length estimation, although his study was limited by the inclusion of a small number of extreme premature and/or ELBW neonates. This could mean that gestational age data is mainly relevant for ELBW neonates.

Chest radiograph remains the gold standard for adequate tube placement confirmation [30]. Although other technical methods have shown promise (i.e., ultrasonography), they require special expertise or are not as widely and/or as readily available [31].

The tube has to be fixated ensuring minimal possible movement with the patient's head kept at a neutral position [21]. Several methods for tube fixation have been reported, but there is currently insufficient evidence to indicate a particular method over another [32].

## 3. Positioning the Patient

In neonates undergoing mechanical ventilation, it has been observed that positions other than the standard supine position, such as the prone position, may improve respiratory performance. The benefits of these positions have not been clearly defined for critically ill neonates receiving MV. A meta-analysis by Rivas-Fernandez et al., including 19 trials involving 516 participants concluded that evidence of low to moderate quality favours the prone position for slightly improved oxygenation in neonates undergoing mechanical ventilation. However, no evidence was found to suggest that particular body positions during mechanical ventilation of the neonate are effective in producing sustained and clinically relevant improvement [33]. There may be additional benefit in raising head of bed slightly to allow expansion of the lungs. This position should be changed periodically to avoid pooling of secretions at base of the lungs [33].

Although with no definitive answer, preterm infants, because of the risk of germinal matrix-intraventricular hemorrhage and the effect of head position on jugular venous drainage, should lie with the head in midline position, at least when the risk of hemorrhage is greatest, that is, during the first three days of life [34].

## 4. Respiratory Kinesiotherapy

The neonatal patient has physiological characteristics such as difficult airway maintenance and clearance, smaller airway caliber, few collateral airways, compliant chest wall, poor airway stability, and lower functional residual capacity [35, 36]. A small amount of mucus can create a large increase in airway resistance, which decreases air flow and, without expiratory flow, the secretions are not expelled. Some respiratory kinesiotherapy maneuvers may allow enough expiratory flow without leading to airway closure [35]. The presence of an endotracheal tube is associated with compromised airway clearance, hinders the cough reflex, impairs mucociliary function, and may increase mucus production [35, 36]. Maneuvers performed periextubation showed improvement in pulmonary symptoms with a decrease in the incidence of lung atelectasis after extubation [35, 36]. Respiratory kinesiotherapy techniques result in lung mechanical effects, improve ventilation, and facilitate the elimination of secretions to avoid bronchial obstruction, allowing good maintenance of airways and facilitating ventilator weaning [37, 38]. However, some authors report that there is not enough evidence to determine whether active chest physiotherapy is of benefit for neonates on mechanical ventilation. Respiratory kinesiotherapy in neonates traditionally includes a multiplicity of techniques: positioning, postural drainage, active techniques such as vibration and percussion, and suction [39]. More recently, other respiratory rehabilitation techniques such as hyperinflation have been applied [35]. Each has indications and contraindications and they are usually used in combination so it is difficult to determine the exact effect of each particular technique [39]. Further studies are still needed to address this issue. Neonatal chest manipulation is not risk free and should only be performed by trained personnel with a high level of expertise [35]. Sessions should be short and should precede feeding [38].

## 5. Nursing Care

Nurses caring for newborns receiving mechanical ventilation face several challenges. Observing the monitor, ventilation devices, oxygen delivery systems, and patient's oxygenation along with the patient himself are essential components of nursing care. Highly sensitive equipments are helpful for the monitoring of the patient. Alarm limits (upper and lower) for heart rate, respiration, blood pressure, and oxygen saturation are set on the basis of current evidence and the NICU's specific standard of care [40].

Expertise and extreme care are important aspects in providing safe and effective nursing care. Cardinal aspects of care include thermoregulation, optimal positioning, airway clearance, stable hemodynamic status, and adequate nutrition for maintenance of growth and development [41].

Open, honest communication with the family is necessary for reducing their anxiety [42].

Nurses must provide a safe environment for the infants in the unit and regulate the infection control policies [40].

Hand washing, isolation, surveillance, and screening of visitors are relevant aspects to nurses in the NICUs [40].

Kangaroo care offers benefits to stable ventilated infants, as long as the procedure is safely practiced according to nursing protocols of transfer from and back to the incubator [43, 44].

## 6. Heating and Humidification of the Inspired Air

Intubation eliminates the natural mechanisms of filtration, humidification, and warming of inspired air [45].

Air inspired to the nose is warmed and nearly 90% humidified by the time it passes through the pharynx [45] The administration of dry oxygen lowers the water content of the inspired air, and the use of artificial airway bypasses the nasopharynx and oropharynx where the humidification of gases primarily takes place [46].

If humidification of inspired gases is not appropriately addressed, ciliary dysfunction, inflammation and necrosis of the ciliated pulmonary epithelium, retention of dried secretions, atelectasis, bacterial infiltration of the pulmonary mucosa, and pneumonia may occur. A humidifier warms and humidifies the gases delivered to the infant during mechanical ventilation via the inspiratory line of the ventilator circuit [47].

Humidifiers are classified as active if they have external sources of heat, water, and flow. The bypass humidifiers are the most widely used today in the NICUs. The gas that goes to the infant passes over the surface of the heated water [48]. Sophisticated systems composed of reservoirs, wires, heating devices, and other elements are of common use in the intensive care unit [49].

## 7. Aerosolization and Nebulization

Aerosol therapy has proven to be an effective form of drug delivery. Despite routine use in the NICUs, the development of appropriate devices as well as medical agents still presents a challenge [50, 51].

Equipment for aerosolization in neonates include inhalers (metered dose inhalers (MDI)), holding chambers, and nebulizers (ultrasonic, jet and vibrating mesh nebulizers, and capillary aerosol generator) [50–52]. Examples of drugs used in critically ill neonates are surfactants, corticosteroids, bronchodilators, diuretics, and antiviral and vasoactive agents [50, 51].

The particular characteristics of neonatal population (low tidal volumes and functional residual capacity, high respiratory rate, a shortened particle residence time, and smaller airway diameters) account for the diminished delivery of inhaled aerosol [50, 51]. Effectiveness of inhalational therapies is influenced by numerous other factors, including the device itself, type and location of the nebulizer, aerosol characteristics (particle size, shape, and density), ventilation gas conditions (flow and humidity), patient interface, and mode of breathing support [50–53].

Current recommendations for the conventionally ventilated neonate (SIMV, AC, VG, and VC) and for those who are on CPAP (continuous positive airway pressure) aim to clean the artificial airways before nebulization, remove the ventilator flow sensor from the wye connector, use vibrating mesh nebulizer (Aeroneb, Pari e-flow) or MDI with holding chamber, place the device in the inspiratory limb in the circuit 20 cm from the wye connector, and optimize breathing support (increase inspiratory time as much as possible, decrease respiratory rate as much as possible, bypass the humidifier, and maintaining air flow heating during nebulization) [53].

## 8. Prevention of Infection

Infection, and its prevention, is an important concern in neonatal ventilated patients, particularly those of very low birth weight. Ventilator-associated pneumonia (VAP), defined as a lung infection diagnosed in a mechanically ventilated patient for >48 h [54], is the second most common form of nosocomial infection and a common device associated complication that occurs at a variable rate of 2.3 to 10.9 episodes per 1000 ventilator days in developed countries [55–57].

There are no universally accepted criteria to diagnose VAP in the neonatal period [58]. Less than 12-month-old infant *Center of Disease Control* VAP diagnostic guidelines have been used in neonates, according to which, in order to be diagnosed with VAP, the ventilated patient has to fulfil clinical (at least one of the following: presence of tachypnea, apnea, and/or retractions; increased need of supplemental oxygen, respiratory settings to achieve targeted respiratory values, amount of respiratory secretions, incidence of desaturation events), radiological (persistent infiltrates or consolidation in two sequential radiographs after the initiation of mechanical ventilation), and microbiological criteria (isolation of a microorganism obtained by bronchoalveolar lavage or isolation of a microorganism in blood culture without any other focus or histopathological examination) [59, 60]. Microbiological study of tracheal aspirates is not reliable for the diagnosis of VAP since airway colonization cannot be dismissed by this technique. The standard for microbiological sampling of the airway is bronchoscopic bronchoalveolar lavage and protected specimen brush, whose invasive character precludes its universal use in neonates intubated with small diameter tubes for whom blind-protected bronchoalveolar lavage should be applied [61–65].

Several risk factors have been associated with the occurrence of VAP. Of these, duration of mechanical ventilation and ELBW infants seem to be the most significant in multivariate analysis [60, 61], although others like length of hospital stay, reintubation, enteral feeding, mechanical ventilation, transfusion, low birth weight, prematurity, bronchopulmonary dysplasia, and parenteral nutrition have been identified in a recent meta-analysis of observational studies [62]. On the contrary, decreasing the frequency of ventilator circuit changes from every seven to 14 days does not seem to influence the VAP rate [62].

The most common agents involved in VAP are Gram-negative bacteria (particularly *Pseudomonas aeruginosa, Enterobacter* species, and *Klebsiella* species) although Gram-positive bacteria, namely, coagulase negative staphylococci and *Staphylococcus aureus*, also play a role [61, 62, 64, 65]. Polymicrobial cultures are frequently found when tracheal aspirate sampling is used. There is no consensus for the initial treatment of VAP. Initial empirical treatment should include broad spectrum antibiotics with coverage for Gram-positive and Gram-negative bacteria, based on likely causative agents and local antimicrobial resistance patterns [65].

VAP is associated with increased length of hospital stay and mortality [61]. In the *Neo-Kiss* registry, Leistner and coworkers found that VAP incidence may influence mortality rate in infants with birth weight between 1000 g and 1500 g [56], in accordance with results from other groups of researchers [64].

Neonatal VAP prevention bundles implementation has been shown to decrease the rate of VAP in NICUs [64]. Such bundles, which apply several evidence-based practices at the same time, have proved to result in greater practice improvements than the sum of the benefits of each practice on its own [66]. They should include practices relative to head position in the ventilated neonate, the use of closed multiuse suction catheters, frequency at which suctioning systems should be changed, routine changing of breathing circuits, assessment of readiness for extubation and cautious evaluation of the need for reintubation, use of medications that interfere with gastric acidity, use of antibiotic bowel decontamination and oral hygiene, and use of separate oral and tracheal suctioning equipment [66]. As with other healthcare-related infections, improvement of caregiver education and hand hygiene remains a very important measure to control VAP incidence.

## 9. Sedation and Analgesia

It is now known that preterm infants are more sensitive to pain than older children and that intubation and invasive mechanical ventilation have physiologic changes determining stress and pain, which can be reduced with sedatives and analgesics [67]. Nowadays, the prevention and treatment of pain and distress represents an essential component of clinical practice [67, 68]. A recent study, including 18 European NICUs, showed that only 46% of invasively ventilated neonates received assessment of continuous pain [69]. Another study documented a generally widespread, but still highly variable use of analgesia/sedation and pain assessment practices at Italian NICUs, despite the diffusion of national guidelines, showing that there is an urgent need to improve routine pain assessment and stress and pain control/prevention in all infants [70].

After the correct pain assessment, the treatment of pain and stress should be done. Nonpharmacological interventions such as nonnutritive sucking and sucrose must be tried. Their use in extremely preterm, unstable, ventilated neonates needs to be addressed [71].

Routine administration of sedation or analgesia in preterm neonates is not recommended due to the safety concerns regarding the pharmacotherapy. Nevertheless, preterm newborns who remain ventilated can be under morphine. Rarely fentanyl or remifentanil should be used [71].

We focus in the sedative and analgesic medication usually used in NICUs: midazolam, morphine, and other opioids, namely, fentanyl and remifentanil (Table 2) [67, 72–77].

In the delivery room, nasal midazolam was more efficient than ketamine to adequately sedate neonates requiring intubation. The hemodynamics and respiratory effects of both drugs were comparable [78]. Preterm infants receiving MIST (minimal invasive surfactant therapy) were more comfortable when sedation was given, but needed ventilation more often [79]. A randomized controlled trial is necessary to test whether the benefit of sedation outweighs the risks of complications.

A neonatal pain and sedation protocol should be implemented in each NICU. It can increase the opiate exposure, but seems not to affect neurodevelopmental outcomes of extremely preterm infants [80]. However, as with any medication, the possibility of short- and long-term adverse reactions must be considered. Nonpharmacological therapy should be used as much as possible [81, 82].

Neuromuscular blocking agents are not recommended in the NICU. A transient curarization can be used during brief diagnostic or therapeutic procedures in order to avoid hemodynamic consequences of deep sedation [83].

## 10. Weaning and Extubation

Weaning and extubation shifts the work of breathing from ventilator to patient, and respiratory drive must be adequate to sustain alveolar ventilation with a tidal volume of 4 ml/kg [84]. Sedation should be reduced and stopped if possible before extubation [85]. As patient's ventilation improves, FiO_2_ should be reduced to below 0.40 and ventilatory parameters can be decreased along with normal blood gases, and one should decrease the most harmful parameter first and each at a time [86]. Methylxanthines are helpful in the preterm neonate, and these populations should be started on nasal CPAP or high flow nasal cannula after extubation [86]. Systemic steroids and diuretics may be useful to extubate preterms still ventilated after the first week of life [86]. Anemia may need to be corrected and a hemoglobin over 15 g/dl is indicated in selected cases [86]. Prone positioning can be helpful in stabilizing the chest wall and improving diaphragmatic excursion. A chest radiograph is not routinely necessary, unless there is clinical evidence of respiratory distress [86].

## 11. Tracheostomy

Neonatal tracheostomy is a common need of newborns requiring prolonged ventilation. Studies have shown that early tracheostomy reduces the incidence of subglottic and tracheal stenosis in children who are intubated for long periods and results in improved comfort, decreased need for sedation, systemic corticosteroid, improved nutrition and growth, ability to attempt oral feeds, and, once established, vocalization with a speaking valve. Improved survival of extremely low and very low birth weight and medically complex infants can result in prolonged mechanical ventilation and sometimes tracheostomy [87].

The indications for neonatal tracheostomy have changed over time. With the need for long-term ventilation, it has become more common [88]. Overman et al. performed a retrospective review of 165 infants who required tracheostomy and ventilator support with a median gestational age of 27 weeks (range 22–43), and birth weight was 820 g (range 360–4860). Overall tracheostomy rate was 6.9% (87/1345) for infants with birth weight ≤1000 g versus 0.9% (64/6818) for infants >1000 g (*P* < 0.001). Median day of life of tracheostomy was 112 (18–573) for infants ≤1000 g and 57 (1–385) for infants >1000 g (*P* < 0.001). Infants ≤1000 g had a longer time from intubation to positive pressure ventilation independence, 505 days (range 62–1287) versus 372 days for >1000 g (range 15–1270; *P* = 0.011) [88].

Common indications for neonatal and infant tracheostomy include congenital or acquired airway obstruction and chronic medical conditions (cardiac disease, neuromuscular disease, and bronchopulmonary dysplasia). Decisions about tracheostomy in neonates involve careful consideration of a number of factors. Mortality, potential short- and long-term outcomes, prospects for home ventilation therapy, and alternatives to tracheostomy should be considered [89].

The specific technique of neonatal tracheostomy varies little from a well-performed tracheostomy and should be performed in the operating room [90]. Tracheostomy has the potential for significant morbidity. Meticulous technique, surgeon experience, and specialized care may play a role in reducing the complication rate. However, complications are usually minor in neonates and do not require additional surgical interventions [91].

## 12. Ventilation in Palliative Care

Despite advances in neonatal medicine and intensive care, there are some infants whose treatments are harmful, are not or are no longer beneficial, and may be discontinued after discussion with the family [92]. The aim of palliative care is to keep the baby comfortable and to support the parents in caring for their baby according to their wishes and beliefs [93, 94].

The process of withdrawal includes the explanation to the parents what will happen; which member of staff will be responsible for the actual removal of the endotracheal tube and turning the ventilator off; the aspiration of the nasogastric tube (considering not feeding the infant just prior to extubation); the alarms of the ventilator and monitors should be turned off prior the disconnection; the endotracheal tube should be suctioned before removal; the parents should be given the choice of being present and holding their infant. Withdrawal of less invasive forms or respiratory support such as nasal continuous positive airway pressure and nasal cannula oxygen may be appropriate if a baby is dying and continued provision of respiratory support only serves to delay death [92–95]. Neuromuscular blocking agents should never be introduced when the ventilator is being withdrawn. If the newborn has been on paralytics, these should ideally have been weaned off hours to days earlier [93]. Analgesics and sedatives should be titrated to relieve pain. It is common for children to experience increased pain, agitation, and dyspnea after extubation, requiring increased doses of medication [92]. In some cases, it is not necessary to turn off the ventilator or extubate the neonate, the parameters of ventilation can be decreased until reaching minimum values, with a decrease of oxygen. Noninvasive ventilation can be used to relief signs of respiratory distress [93].

## 13. Care in ECMO

ECMO (extracorporeal membrane oxygenation) is a treatment that allows to provide cardiorespiratory support. The veno-venous (VV) ECMO does so by increasing the oxygen content in the venous blood. Veno-arterial (VA) ECMO, in addition to increasing blood oxygen content, may provide hemodynamic support by increasing systemic blood flow. This technique, in the presence of pulmonary pathology, should allow the recovery of pulmonary structure and function in order to ensure the survival of the newborn [96, 97].

After initiating VA-ECMO, ventilatory parameters can be rapidly weaned. The patient should remain during the treatment (to allow recovery of lung function) with “rest” settings (FiO_2_ 25–30%, peak inspiratory pressure (PIP) 20–25 cm H_2_O; positive end expiratory pressure (PEEP) 4-5 cm H_2_O; inspiratory time (Ti) 0.5–1.0 seconds; frequency (F) 20–25 breaths per minute) or even in continuous positive airway pressure (CPAP) with a high PEEP (10–12 cm H_2_O) in order to maintain functional residual capacity (FRC) without causing lung injury (volutrauma, barotrauma, or atelectrauma). In VA-ECMO, it is essential to integrate the presence of two lungs and two hearts functioning in parallel. Improvement of native cardiac function may translate into a reduction in arterial oxygen saturation, but with increased tissue oxygen delivery.

After initiating VV-ECMO, weaning from ventilatory and hemodynamic support should be done slowly and with caution, since oxygen delivery is dependent on native myocardial function and, on the other hand, the native lung still provides gas exchanges. The use of a high PEEP may compromise pulmonary blood flow and cardiac output. The resting parameters to be achieved will be the same as for VA-ECMO, but we should use expired CO_2_ to optimize PEEP [98–100].

During ECMO treatment, mild and protective ventilatory strategies can and should be employed, without concern for ventilation/oxygenation, which is ensured by the technique employed [100].

In acute technical failure (e.g., accidental decannulation) rescue parameters must be clearly indicated and should be immediately set on the ventilator according to the clinical condition of the patient [99].

## 14. Complications: Air Leaks and Pulmonary Hemorrhage

Air leak syndrome refers to the extravasation of air from the tracheobronchial tree into the lung parenchyma and pleural spaces where it is not normally present, and includes pneumothorax, pulmonary interstitial emphysema, pneumomediastinum, pneumopericardium, pneumoperitoneum, subcutaneous emphysema, and systemic air embolism.

Air leak is a common complication of mechanical ventilation, occurring in up to 40% of ventilated newborns [101]. Risk factors include prematurity, very low birth weight, low Apgar score, high peak inspiratory pressure, high tidal volume, high inspiratory time, respiratory distress syndrome, meconium aspiration syndrome, amniotic fluid aspiration, pneumonia, and pulmonary hypoplasia [102–104].

The most frequent air leak in mechanical ventilated newborns is pneumothorax. Different ventilatory strategies affect the risk of pneumothorax with evidence that high-frequency ventilation [105], volume-targeted ventilation [106], and increased PEEP [104] are associated with a decreased risk, and continuous positive inspiratory pressure is associated with an increased risk of pneumothorax [107, 108].

The clinical presentation ranges from asymptomatic to severe progressive respiratory distress [102] and, in case of tension pneumothorax, hemodynamic compromise [109]. Physical examination may reveal tracheal deviation, asymmetrical chest rise, diminished breath sounds over the affected side, and muffled or shifted heart sounds [109]. Diagnosis is usually made by radiography [109]. However, in neonates, the classic appearance may be more difficult to recognize. One study evaluating the role of special radiological signs in the diagnosis of pneumothorax in neonates found that 46% of the neonates with pneumothorax had special radiological signs 0.5 to 27 hours before they were clinically diagnosed, namely, deep sulcus sign, medial stripe sign, basilar hyperlucency, increased cardiomediastinal sharpness, large hyperlucent hemithorax, and the double diaphragm sign [110]. Therefore, these signs should be looked for in ventilated newborns in order to perform an early diagnosis of pneumothorax.

A tension pneumothorax requires immediate diagnosis and intervention even before imaging is obtained, and chest transillumination plays a role in these cases [101, 109].

Treatment options include conservative management, nitrogen washout with oxygen, needle aspiration, intercostals tube drainage, and placement of a pigtail catheter. A study including only adult patients reports a rate of spontaneous reabsorption of pneumothorax of 1.2% of the volume of the hemithorax per 24 hours [110]. An expectant management may be effective even in neonates undergoing mechanical ventilation [111, 112], but intervention is most often needed [111].

Placing the infant under high concentrations of oxygen may hasten the reabsorption of gas in the pleural space by creating a diffusion gradient. However, in neonates, this technique is limited by the oxygen toxicity and increased risk of retinopathy of prematurity [109].

Needle thoracentesis with aspiration is the preferred treatment in emergencies such as a tension pneumothorax, but may not completely correct the situation [109, 111]. The most traditional method of treatment of a pneumothorax is a chest tube placed by thoracostomy [109, 112, 113]. However, a recent systematic review found insufficient evidence to determine the efficacy and safety of needle aspiration versus intercostal tube drainage in the management of pneumothorax in newborns [114].

More recently, pigtail catheters have been used for treatment of pneumothorax in newborns, with evidence to be safe, effective, and reduce discomfort during insertion and complications [109] including in preterm neonates [115].

Further prospective randomized controlled trials are necessary to determine which method is superior in the management of neonatal pneumothorax in mechanical ventilated patients.

Pulmonary hemorrhage is a rare but severe condition, which is characterized by massive bleeding within the lung parenchyma and airways, with a mortality above 50% during the neonatal period [116]. The most consistent risk factors for its occurrence are prematurity, respiratory distress syndrome (RDS), the use of exogenous surfactant, and a patent ductus arteriosus (PDA), especially in premature infants less than 28 weeks gestational age and/or birth weight less than 1000 grams. Other risk factors include pulmonary interstitial emphysema (PIE), pneumothorax, pulmonary infection, metabolic acidosis, shock, hypothermia, hypoglycemia, disseminated intravascular coagulation (DIC), ECMO therapy, hereditary coagulation disorders, and airway trauma (especially following endotracheal intubation) [117, 118].

Pulmonary hemorrhage clinically presents with a rapidly worsening pulmonary function (the speed of the setting depends obviously on the magnitude of the hemorrhage), with hypoxia, hypercarbia, and the need for increased ventilatory parameters. Blood can be seen in oropharyngeal or tracheal aspirates. A systemic deterioration is established with metabolic acidosis and shock. Investigations should include a chest X-ray, an echocardiogram (to exclude a PDA), work-up for sepsis, and eventual screening for hereditary diseases of coagulation (if no other risk factors are detected) [119].

Treatment should include general supportive measures: transfusions of blood, plasma or platelets, as indicated; correction of metabolic acidosis; inotropic drugs to improve systemic blood pressure; PDA treatment (except severe thrombocytopenia); antibiotic treatment including vancomycin; and coverage for Gram-negative bacteria [118, 119].

The ventilatory strategy employed is essentially based on empirical observations, without recommendations based on evidence. In conventional ventilatory support, the increase in settings is recommended (higher frequency (F), higher positive end expiratory pressure (PEEP), and higher mean airway pressure (Pāw)). Although without evidence, observational studies suggest that the support with high-frequency oscillatory ventilation (HFOV) allows to control more cases of pulmonary hemorrhage when comparing to conventional support [118, 119].

Other specific therapeutic measures include treatment with recombinant factor VIIa (dose, frequency, and consistency of response not yet established in neonatal patients), nebulized epinephrine, and repeated instillations of epinephrine (0.5 mL of 1 : 10,000 dilution) by the endotracheal tube [118].

## 15. Monitoring of the Ventilated Neonate

Along with respiratory care for the ventilated neonate, ventilation monitoring is of great importance for a good oxygenation avoiding hypercapnia and hypocapnia, both associated with deleterious effects not only on preterm but also on term brain. Pulse oximetry, pH and blood gases measurements, transcutaneous carbon dioxide measurement, and capnography are standard of care in most actual NICUs [120]. Capnography is a standard tool in mechanically ventilated adult and pediatric patients, but it has physiological and technical limitations in neonates. The high respiratory rate and low tidal volume in neonates require main-stream sensors with fast response times and minimal dead-space or low suction flow when using side-stream measurements. These technical requirements are difficult to fulfil in neonates and the measured end-tidal CO_2_ (Pet CO_2_), which should reflect the alveolar CO_2_ is often misleading, and capnography is mostly used to evaluate the trend of Pet CO_2_ [121].

Online pulmonary function and mechanics testing are currently valuable tools to aid clinical decision making in the management of ventilated infants. They are a tool for assessment of patient status, therapeutic evaluation, and management guidance of infants on ventilator. The knowledge of pulmonary graphics also improves understanding of pulmonary physiology and pathophysiology and their responses to mechanical ventilatory support [122, 123]. The new ventilators now provide such information and the clinicians should be familiar with.

Neonatal echocardiography is a valuable and increasingly used tool in the NICU and can contribute substantially to hemodynamic management of the ventilated neonate. It has a major role in excluding a congenital heart defect, in the evaluation of pulmonary hypertension, in the evaluation of cases of hypotension or shock, and the response to therapy, as well as in the management of a patent ductus arteriosus [124].

NIRS measures the regional cerebral saturation in oxygen and may provide an early alert of low levels of cerebral blood flow and brain oxygenation, potentially helping in preventing intraventricular hemorrhage or periventricular leukomalacia in the neonate [125].

Lung ultrasound is an emerging tool of diagnosis and can be of added value helping in monitoring due to the specific pathology inherent in lung immaturity as well as in the particular sensitivity of neonates to repeated radiation exposure.[126].

## 16. Conclusion

In conclusion, we can say that invasive ventilation is often necessary for the treatment of newborn infants with respiratory insufficiency, and the neonatal patient has unique physiological characteristics. Each clinical situation imposes a global attention to the overall clinical status of the patient and associated comorbidities. Respiratory care has to be individualized and needs to be adapted to patient's characteristics, the clinical condition, associated comorbidities, and the overall prognosis. The importance of this review is to highlight important aspects to be taken in during care of the ventilated newborn in order to optimize patient ventilation and monitoring, simultaneously without causing lesions possibly resulting from inadequate ventilation.

## Figures and Tables

**Table 1:** Endotracheal tube insertion depth for oro- and nasotracheal intubation in neonates [28].

Gestational age (weeks)	Current weight (kg)	Endotracheal tube length at lips (cm)	Endotracheal tube length at nostril (cm)
23–24	0.5–0.6	5.5	6.5
25–26	0.7–0.8	6.0	7.0
27–29	0.9–1.0	6.5	7.5
30–32	1.1–1.4	7.0	8.0
33–34	1.5–1.8	7.5	8.5
35–37	1.9–2.4	8.0	9.0
38–40	2.5–3.1	8.5	9.5
41–43	3.2–4.2	9.0	10.0

**Table 2:** Sedative and analgesic medication usually used.

Midazolam	Widely used in NICUs, some decades ago. The usual dose infusion is 10–60 *μ*g/kg/hour. In very preterm infants a high incidence of hypotension and intraventricular hemorrhage have been reported and contraindicate its use [67]. Nowadays, however, data are insufficient to promote the use of intravenous midazolam infusion as a sedative for newborn infants undergoing intensive care [72]. Recently midazolam exposure was associated with structural alterations in hippocampal development and poorer outcomes consistent with hippocampal dysmaturation [73]. Data show that further research on the effectiveness and safety of midazolam in neonates is needed before its use routinely in NICUs.

Morphine	Has been used in low-dose infusion (10 *μ*g/kg/h) to control stress in mechanically ventilated infants. Opioids prolong mechanical ventilation due to the suppression of the respiratory drive [67]. However, due to the lack of available alternative drugs, they are used in clinical practice in neonatology with marked variability among NICUs [74]. Although much is known about morphine in newborns, there is insufficient evidence to recommend routine use of opioids in mechanically ventilated newborns. Opioids should be used selectively, by clinical judgment and evaluation of pain. If sedation is required, morphine is safer than midazolam [75, 76].

Fentanyl	Infusion is usually of 1.5 *μ*g/kg/h. It can induce chest wall rigidity and laryngospasm in both term and preterm newborns. There is a significant accumulation due to prolonged half-life in preterm neonates, even with low doses. So, continuous infusion of fentanyl should be avoided in preterm infants [67].

Remifentanil	Doses of 1–4 *μ*g/kg showed to be as effective as the morphine-midazolam regimen for endotracheal intubation. The rapid administration of remifentanil provides insufficient sedation and is associated, like fentanyl, with a chest wall rigidity in preterm neonates [77].
